# Roles of the *rpoEc-chrR-chrA* operon in superoxide tolerance and β-lactam susceptibility of *Stenotrophomonas maltophilia*

**DOI:** 10.3389/fcimb.2025.1492008

**Published:** 2025-02-04

**Authors:** Ren-Hsuan Ku, Hsu-Feng Lu, Li-Hua Li, Ting-Yu Yeh, Yi-Tsung Lin, Tsuey-Ching Yang

**Affiliations:** ^1^ Department of Biotechnology and Laboratory Science in Medicine, National Yang Ming Chiao Tung University, Taipei, Taiwan; ^2^ Department of Medical Laboratory Science and Biotechnology, Asia University, Taichung, Taiwan; ^3^ Department of Pathology and Laboratory Medicine, Taipei Veterans General Hospital, Taipei, Taiwan; ^4^ School of Medical Laboratory Science and Biotechnology, College of Medical Science and Technology, Taipei Medical University, Taipei, Taiwan; ^5^ Division of Infectious Diseases, Department of Medicine, Taipei Veterans General Hospital, Taipei, Taiwan; ^6^ School of Medicine, National Yang Ming Chiao Tung University, Taipei, Taiwan

**Keywords:** *Stenotrophomonas maltophilia*, singlet oxygen, β-lactam susceptibility, oxidative stress, sigma factor

## Abstract

**Introduction:**

The *rpoE-chrR* pair is a regulatory system used by photosynthetic microorganisms to overcome singlet oxygen stress. *rpoE* and *chrR* encode the sigma factor σ^E^ and anti-sigma factor ChrR, respectively. Stenotrophomonas maltophilia, an opportunistic pathogen, is a multidrug-resistant gram-negative bacterium. Although it is not a photosynthetic microorganism, a *rpoE-chrR* homolog (*smlt2377-smlt2378*) was found in the *S. maltophilia* genome. In this study, we aimed to assess the significance of σ^Ec^-ChrR pair in oxidative stress alleviation and antibiotic susceptibility of *S. maltophilia* KJ.

**Methods:**

Reverse transcription-polymerase chain reaction was used to validate the presence of operon. The contribution of *rpoEc-chrR-chrA* operon to oxidative stress alleviation and antibiotic susceptibility was evaluated using mutant constructs and stress-tolerance assays. RNA-seq transcriptome assay of wild-type KJ, KJΔChrR (*chrR* mutant), and KJΔChrRΔRpoEc (*chrR/rpoEc* double mutant) was performed to reveal the σ^Ec^ regulon.

**Results:**

The *rpoEc-chrR* pair and downstream chrA formed an operon. Inactivation of *chrR* upregulated the expression of *rpoEc-chrR-chrA* operon in an σ^Ec^- and ChrA-dependent manner. σ^Ec^ activation contributed to superoxide tolerance and increased β-lactam susceptibility but did not affect the tolerance to singlet oxygen and hydrogen peroxide. Transcriptome analysis revealed that expression of the nine-gene cluster, *smlt2375-smlt2367*, was significantly upregulated in KJΔChrR and reverted to the wild-type level in KJΔChrRΔRpoEc. *smlt2375-smlt2367* cluster was located upstream of the *rpoEc-chrR-chrA* operon and divergently transcribed, seeming to be involved in membrane lipid modification. Deletion of *smlt2375-smlt2367* cluster from the chromosome of KJΔChrR reverted the superoxide tolerance and β-lactam susceptibility to the wild-type level.

**Discussion:**

The *rpoEc-chrR* pair of *S. maltophilia* was involved in superoxide tolerance and β-lactam susceptibility. Notably, a novel regulatory circuit involving *rpoEc-chrR-chrA* operon and *smlt2375-smlt2367* cluster was revealed.

## 1 Introduction

Pathogens frequently encounter various stressors imposed by host cells, such as iron depletion, reactive oxygen species (ROS), and envelope-damaging agents (Sun and Zhou, 2018). Bacteria have evolved various mechanisms to deal with such environmental stresses (Marles-Wright and Lewis, 2007). One mechanism involves the reversible association of different σ factors with the bacterial core RNA polymerase (RNAP) to control the expression of discrete sets of genes in response to stress-related signals (Helmann, 2002).

RNAP is an essential transcriptional enzyme. Bacterial core RNAP has five subunits: α^I^, α^II^, β, β‘, and ω. The sigma (σ) factor binds to the core RNAP to form a holoenzyme. Sigma factor is a transcription initiation factor that facilitates the specific binding of RNAP to gene promoters. Bacteria usually have one housekeeping σ factor (σ^D^) and an array of alternative σ factors possessing different promoter-recognition properties to regulate the subsets of genes necessary for survival under specific environmental conditions or stress responses (Gruber and Gross, 2003). The number of σ factors varies among bacteria, possibly related to their habitat and metabolism. The sigma factors are classified into two distinct families: σ^70^ and σ^54^. The σ^70^ family members are further divided into four groups: group 1 comprising primary factors and groups 2–4 consisting of alternative factors with specialized functions (Davis et al., 2017).

Extracytoplasmic function (ECF) sigma factors, the largest and most diverse group of alternative sigma factors, belong to group 4 of the σ^70^ families (Mascher, 2023). Several mechanisms responsible for the regulation of ECF sigma factor activity have been described. In general, these activities are inhibited by anti-σ factors. In response to stimuli, anti-σ factors use diverse mechanisms to release σ factors that then bind to the core enzyme and drive the transcription of an array of genes. The subset of genes regulated in this manner is referred to as the regulon. The genes encoding ECF σ factor and anti-σ factor are generally organized in an operon (Paget, 2015). The most representative model is the RpoE/RseA model of *Escherichia coli*. RseA, an anti-σ^E^ factor, is an integral cytoplasmic membrane protein with a C-terminal extracytoplasmic sensory domain and an N-terminal intracellular inhibitory domain that binds to cognate σ^E^ protein. Upon challenge with stimuli, RseA is subsequently hydrolyzed by a two-step intramembrane proteolysis, and σ^E^ is released to trigger the transcription of responsive genes (Ho and Ellermeier, 2012). In addition to the inner membrane anti-σ^E^ factor, an example of cytoplasmic anti-σ factor is discovered in the phototrophic alpha-proteobacterium *Rhodobacter sphaeroides*. In this microorganism, σ^E^-ChrR pair is a regulatory system to alleviate the singlet oxygen stress. Under unstressed conditions, σ^E^ binds to the anti-σ factor ChrR, which maintains σ^E^ in an inactive state. Singlet oxygen acts as a signal that is sensed in an unidentified manner and leads to σ^E^ release, thereby inducing the expression of σ^E^ regulon to alleviate singlet oxygen stress (Donohue, 2019).

Singlet oxygen, one of the ROS, is the primary agent of photo-oxidative stress in photosynthetic microorganisms. In these microorganisms, singlet oxygen is generated by energy transfer from the excited pigments of the photosystems (Sharma et al., 2012). A singlet oxygen molecule can react with a wide range of cellular macromolecules to cause damage (Triantaphylides and Hayaux, 2009). Interestingly, singlet oxygen is also produced in non-photosynthetic microorganisms because cellular cofactors, such as flavins, rhodopsins, quinones, and porphyrins, serve as photosensitizers. To face the singlet oxygen stress, microorganisms have evolved several mechanisms to alleviate singlet oxygen-mediated stress, including quenchers (such as carotenoids) and scavengers (such as glutathione) (Glaeser et al., 2011).

*Stenotrophomonas maltophilia* is a ubiquitous environmental bacterium that acts as an opportunistic pathogen causing various clinical conditions, mainly pulmonary and bloodstream infections (Sánchez, 2015). From the external to the host environment, *S. maltophilia* is subjected to various stresses, including nutrient unavailability, oxidative stress, osmotic stress, and iron-depletion; therefore, it has evolved various mechanisms to adapt to the dynamic environment (Huang et al., 2014; Lin et al., 2014; Wu et al., 2018; Jair et al., 2019; Huang et al., 2019; Li et al., 2020a, 2020; Pan et al., 2022; Shih et al., 2022; Liao et al., 2022). *S. maltophilia* is intrinsically resistant to several antibiotics as it possesses various determinants contributing to antibiotic resistance, such as β-lactamases, efflux pumps, and aminoglycoside-modifying enzymes (Mojica et al., 2022). These defense mechanisms make the treatment of *S. maltophilia* infections challenging. *S. maltophilia* is intrinsically resistant to most β-lactams due to the chromosomally encoded L1 and L2 β-lactamases. Among β-lactams, ceftazidime (CAZ) and ticarcillin-clavulanic acid are the only ones suitable for treating *S. maltophilia* infections. L1 and L2 β-lactamases inducible expression in *S. maltophilia* is linked to the disturbance of peptidoglycan (PG) homeostasis (Mojica et al., 2022), similar to AmpC expression in *Enterobacter cloacea*, *Citrobacter freundii*, and *Pseudomonas aeruginosa* (Jacoby, 2009). In addition to β-lactamase, non-β-lactamase-mediated β-lactam resistance in *S. maltophilia* has also been reported. For example, loss-of-function of PhoPQ two-component regulatory system increases the outer membrane permeability, which leads to increased β-lactam susceptibility of *S. maltophilia* (Huang et al., 2021).

Some stress-alleviation systems contribute to antibiotic resistance (Shin et al., 2020). We reported that the *smeU1VWU2X* operon, which encodes an RND-type efflux pump, not only alleviates menadione (MD)-mediated oxidative stress but also contributes to antibiotic resistance in *S. maltophilia* (Wu et al., 2018). Furthermore, formaldehyde detoxification system FadRACB participates in the alleviation of oxidative stress and fluoroquinolone resistance in *S. maltophilia* (Li et al., 2020b). A genome-wide survey revealed that *S. maltophilia* K279a harbors the *rpoE-chrR* pair (Crossman et al., 2008), although it is not a photosynthetic microorganism. This observation raised our curiosity about the significance of the *rpoE-chrR* system in *S. maltophilia*. In this study, we aimed to elucidate the role of the σ^E^-ChrR pair of *S. maltophilia* in oxidative stress alleviation and antibiotic susceptibility.

## 2 Materials and methods

### 2.1 Bacterial strains, plasmids, and primers

The strains, plasmids and primers used in this study are shown in **Supplementary Table S1**.

### 2.2 Reverse transcription-PCR and operon verification

DNA-free RNA was prepared from *S. maltophilia* KJΔChrR cells and reverse transcribed into cDNA using the ChrA-C primer (**Supplementary Table S1**). cDNA was used as the template for PCR using the primer sets of RpoEcQ-F/R, ChrRQ-F/R, and ChrAQ-F/R (**Supplementary Table S1**). The ChrAQ-F/R primer sets were used as a control for DNA contamination check.

### 2.3 In-frame deletion mutation construction

In-frame deletion mutants were constructed using allelic replacement strategy as described previously (Yang et al., 2009). Two DNA fragments flanking the deleted region were amplified by PCR and subsequently cloned into pEX18Tc to generate pΔRpoEc, pΔChrR, and pΔChrA. The primer sets used were RpoEcN-F/R and RpoEcC-F/R for pΔRpoEc, RpoEcC-F/R and ChrRC-F/R for pΔChrR, as well as ChrRC-F/R and ChrAC-F/R for pΔChrA (**Supplementary Table S1**). The resulting pEX18Tc-derived constructs were transported to *S. maltophilia* strain by conjugation. Transconjugants selection and mutants confirmation were performed as described previously (Yang et al., 2009). The double and triple mutants were constructed from the single mutant sequentially using the same protocol.

### 2.4 Singlet oxygen tolerance test

Tert-Butyl hydroperoxide (tBOOH) and Rose Bengal (RB) were used for the generation of single oxygen. The tBOOH reacts with peroxynitrite to generate singlet oxygen (Di Mascio et al., 1997). Rose Bengal is a UV/VIS absorbing molecule capable of absorbing and using light energy to excite oxygen to singlet oxygen (DeRosa and Crutchley, 2002). For cell viability, the logarithmic-phase bacterial cells tested of 2 × 10^5^ CFU/μL were 10-fold serially diluted. Five microliters of the bacterial suspension were spotted onto the LB agar with and without tBOOH or RB as indicated. For the RB test, two plates were prepared. One plate was covered with foil seal to create “dark” conditions in which little or no singlet oxygen is produced. The other plate was kept in the light, representing the singlet oxygen-stressed condition. After 18-h incubation at 37°C, the bacterial viability was imaged. For growth curve, overnight culture was inoculated into LB broth with and without tBOOH or RB at an initial OD_450_ of 0.15. Bacterial growth was monitored for 24 h.

### 2.5 Menadione and antibiotic tolerance assay

For cell viability, the logarithmic-phase bacterial cells of 2×10^5^ CFU/μL were serially 10-fold diluted. Five microliter bacterial aliquot was spotted onto LB agar with and without MD or antibiotic as indicated. After a 24-h incubation at 37˚C, the cell viabilities were recorded. For growth curve, overnight culture was inoculated into LB broth with and without MD or antibiotic at an initial OD_450_ of 0.15. Bacterial growth was monitored for 24 h.

### 2.6 Construction of a *rpoEc* promoter–*xylE* transcriptional fusion, pRpoEc_xylE_

For the construction of a transcriptional fusion of the *rpoEc* promoter with a promoterless *xylE* gene, the 356-bp DNA segment upstream of *rpoEc* was PCR-amplified using *rpoEcN-*F/R primer pair (**Supplementary Table S1**) and cloned into pXylE (Chen et al., 2011) to generate pRpoEc_xylE_.

### 2.7 Catechol-2,3-dioxygenase activity determination

C23O, encoded by *xylE*, can convert catechol to an intensely yellow products, which can be spectrophotometrically quantified. One unit of enzyme activity (Uc) was defined as the amount of enzyme that converts 1 nmol of catechol per minute. The specific activity (Uc/OD_450_) of the enzyme was defined as units per OD450 unit of cells. All data were reported from experiments performed in triplicate.

### 2.8 Transcriptome analysis

Overnight cultures of KJ, KJΔChrR, and KJΔChrRΔRpoEc were inoculated into fresh LB broth at an initial OD_450_ of 0.15. After 5-h culture at 37°C, total RNA was prepared for RNAseq transcriptome analysis. RNA isolation, rRNA depletion, adapter-ligated cDNA library, and cDNA sequencing were carried out as described previously (Wu et al., 2022). The sequencing reads were mapped to the genome of K279a (Crossman et al., 2008). The total number of reads per gene was normalized by transcripts per kilobase million (TPM). The RNA-seq data have been deposited in GenBank under BioProject accession numbers SAMN41918898 for KJ, SAMN41918899 for KJΔChrR, and SAMN41918900 for KJΔChrRΔRpoEc.

### 2.9 Quantitative real-time-PCR

RNA was isolated from logarithmical phase bacterial cells and converted to cDNA using the High Capacity cDNA Reverse Transcription Kit (Applied Biosystems) following the manufacturer’s instructions. Real-time PCR was performed using the TaqMan Universal PCR Master Mix (Applied Biosystems) and an ABI Prism 7000 Sequence Detection System (Applied Biosystems). The primer sets used are listed in **Supplementary Table S1**. The 16S rRNA was used to normalize the gene expressions. Fold change was calculated using the ΔΔ*C_T_* method (Livak and Schmittgen, 2001). Three biological replicates were performed.

## 3 Results

### 3.1 Smlt2377/Smlt2378 is a σ^E^/ChrR homolog in *S. maltophilia*

**Figure 1:**
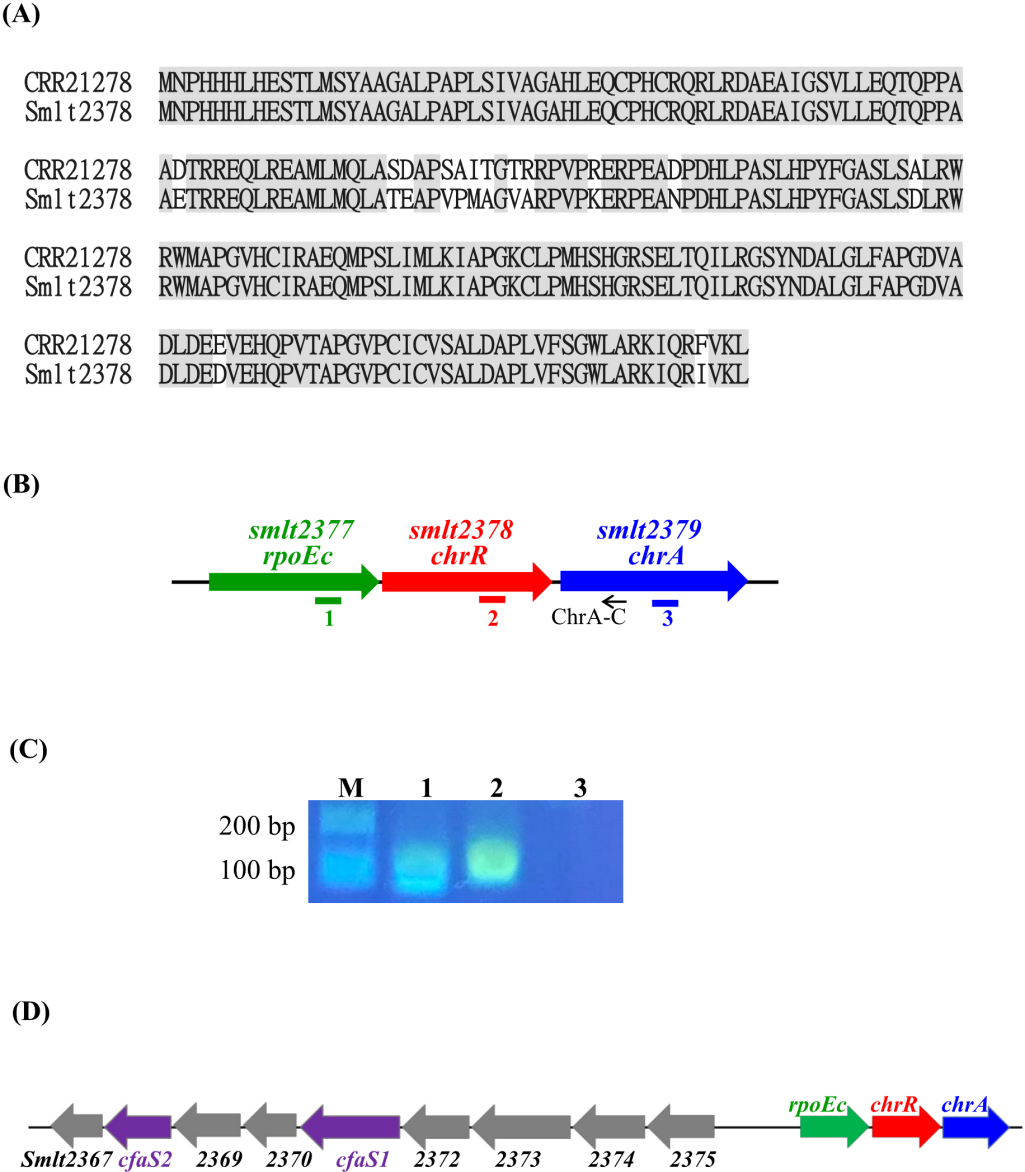
Characterization of *rpoEc-chrR-chrA* operon. **(A)** Protein sequences alignment of *P. aeruginosa* ChrR (accession No. CRR21278) and *S. maltophilia* ChrR (Smlt2378). Protein sequence alignment was carried out using Needleman-Wunsch global alignment. The same amino acid residues are marked in gray. **(B)** Genetic organization of *rpoEc-chrR-chrA* operon. The orientation of the genes is indicated by arrows. The small black arrow indicates the position of the ChrA-C primer used for reverse transcription. Solid bars labeled as 1-3 represent the positions of PCR amplicons correspondent with the labels in **(C)**. **(C)** Verification of *rpoEc-chrR-chrA* operon. Overnight culture of *S. maltophilia* KJΔChrR was inoculated into fresh LB with an initial OD_450_ of 0.15 and grown for 5 h. The cDNAs were obtained by reverse transcription using the primer ChrA-C. cDNA (100 ng) was used as the template for PCR. The PCR amplicons were separated by agarose gel electrophoresis and stained with ethidium bromide. Lane 1, PCR amplicon generated by RpoEcQ-F and RpoEcQ-R; lane 2, PCR amplicon generated by ChrRQ-F and ChrRQ-R; lane 3, PCR amplicon generated by ChrAQ-F and ChrAQ-R. The ChrAQ-F/R primers were used as a control for DNA contamination check. **(D)** Genetic organization of *rpoEc-chrR-chrA* operon and the divergently transcribed nine genes, *smlt2375-smlt2367*.

The σ^E^-ChrR pair homologs are distributed among α-proteobacteria and γ-proteobacteria, such as *R. sphaeroides*, *Vibrionaceae*, and *Pseudomonas* (Glaeser et al., 2011). To identify the σ^E^-ChrR homolog in *S. maltophilia*, we performed BLASTP analysis using *P. aeruginosa* ChrR (accession No. CRR21278) as the query. The search results revealed a single candidate, Smlt2378, showing 94% protein identity with *P. aeruginosa* ChrR (**Figure 1A**). Next, genomic organization surrounding *smlt2378* was surveyed. *smlt2377* encoded an ECF σ factor, demonstrating that the σ^E^-ChrR pair is conserved in *S. maltophilia*. Eleven base pairs downstream of *smlt2378* and in the same orientation, a third open reading frame of 693 bp (Smlt2379) was identified (**Figure 1B**). *smlt2379* encoded a 23.6-kDa cytoplasmic protein with an NAD(P)-binding domain that highly functioned as an oxidoreductase. Genomic organization strongly suggested the presence of the *smlt2377-smlt2378-smlt2379* operon, which was verified via reverse transcription-polymerase chain reaction (RT-PCR) (**Figure 1C**). Based on the results presented in this study, we designated *smlt2377-smlt2378-smlt2379* as *rpoEc-chrR-chrA.* Next, we examined the conservation of *rpoEc-chrR-chrA* operon in *S. maltophilia*. Among the 12 strains surveyed, all harbored this operon, indicating high conservation of the *rpoEc-chrR-chrA* operon in *S. maltophilia*. In addition, we also noticed that there are nine genes (*smlt2375-smlt2367*) located upstream of *rpoEc-chrR-chrA* operon and divergently transcribed (**Figure 1D**). This genomic organization highly suggested that the nine genes may form an operon and regulated by σ^Ec^, which will be further expounded later.

### 3.2 *rpoEc-chrR-chrA* operon barely contributes to singlet oxygen tolerance

**Figure 2:**
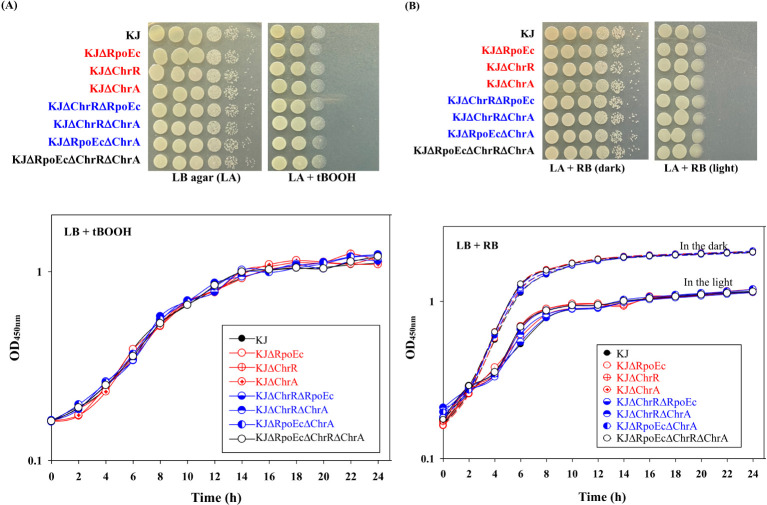
Role of *chrEc-chrR-chrA* operon in singlet oxygen tolerance. **(A)** tBOOH test. For cell viability, the logarithmic-phase bacterial cells tested of 2 × 10^5^ CFU/μL were 10-fold serially diluted. Five microliters of bacterial suspension were spotted onto the LB plates with and without 500 μM tBOOH. After 18-h incubation at 37°C, the bacterial viability was imaged. For growth curve, overnight culture was inoculated into LB broth with 300 μM tBOOH at an initial OD_450_ of 0.15. Bacterial growth was monitored for 24 h. The image and graph are representatives of at least three replicated experiments. **(B)** RB test. For cell viability, the logarithmic-phase bacterial cells tested of 2 × 10^5^ CFU/μL were 10-fold serially diluted. Five microliters of bacterial suspension were spotted onto two LB plates with 300 nM RB. One plate was covered with foil to create “dark” condition. The other plate was kept in the light. After 18-h incubation at 37°C, the bacterial viability was imaged. For growth curve, overnight culture was inoculated into LB broth with 200 nM RB at an initial OD_450_ of 0.15. Both dark and light culture conditions were prepared. Dash and solid lines indicate the cultures in the dark and light, respectively. Bacterial growth was monitored for 24 h. The image and graph are representatives of at least three replicated experiments.

To characterize *rpoEc-chrR-chrA* operon, its genes were deleted from *S. maltophilia* KJ, either alone or in combination, to yield KJΔRpoEc, KJΔChrR, KJΔChrA, KJΔChrRΔRpoEc, KJΔChrRΔChrA, KJΔRpoEcΔChrA, and KJΔRpoEcΔChrRΔChrA (**Supplementary Table S1**). These mutants exhibited no observable growth defects on LB agar (**Figure 2A**) or broth (data not shown).

A study on σ^E^-ChrR pair of *R. sphaeroides* indicated its contribution to the singlet oxygen stress response (Anthony et al., 2004). We were interested in understanding whether the *rpoEc*-*chrR*-*chrA* operon played a role in the alleviation of singlet oxygen stress. Rose Bengal (RB) and tert-butyl hydroperoxide (tBOOH) methods were used to evaluate singlet oxygen tolerance (Di Mascio et al., 1997; Brahmachari and Karmakar, 2020). All the mutants tested displayed almost comparable viability to wild-type KJ in RB- and tBOOH-containing LB agar or broth (**Figures 2A, B**), indicating that *rpoEc-chrR-chrA* hardly contributes to singlet oxygen alleviation in our assay.

### 3.3 *rpoEc-chrR-chrA* operon contributes to superoxide tolerance

**Figure 3:**
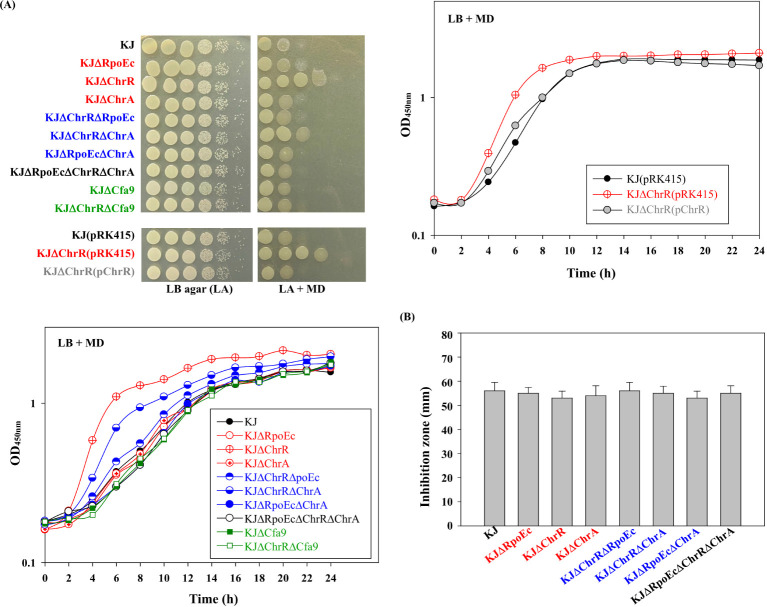
Role of *chrEc-chrR-chrA* operon in MD and H_2_O_2_ tolerance **(A)** Role of *rpoEc-chrR-chrA* operon in MD tolerance. For cell viability, the logarithmic-phase bacterial cells of 2 × 10^5^ CFU/μL were 10-fold serially diluted. Bacterial aliquot (5 μL) was spotted onto LB agars without and with 50 μg/mL MD. After a 24-h incubation at 37°C, the growth of bacterial cells was observed. For growth curve, overnight culture was inoculated into LB broth with 20 μg/mL MD at an initial OD_450_ of 0.15. Bacterial growth was monitored for 24 h. The image and graph are representatives of at least three replicated experiments. **(B)** Role of *rpoEc-chrR-chrA* operon in H_2_O_2_ tolerance. The bacterial cell suspension tested was spread onto LB agar. A sterile filter paper disc with 15 μL 10% H_2_O_2_ was placed on the agar. The growth inhibition zone was measured after a 24-h incubation at 37°C. Bars represent the average values from three independent experiments. Error bars represent the standard deviation for triplicates.

Involvement of the *rpoE_C_-chrR-chrA* operon in superoxide tolerance was assessed using an MD tolerance assay. Inactivation of *chrR* increased bacterial tolerance to MD, and complementation of KJΔChrR with a plasmid containing *chrR* reversed MD tolerance to wild-type levels (**Figure 3A**). Furthermore, further inactivation of *rpoEc* or *chrA* in KJΔChrR partially restored MD tolerance (**Figure 3A**). Thus, σ^Ec^ activation increases MD tolerance, and ChrA is involved in this regulatory circuit.

Next, we investigated the role of *rpoE_C_-chrR-chrA* operon in H_2_O_2_ tolerance. All tested strains exhibited comparable H_2_O_2_ susceptibilities (**Figure 3B**), tentatively ruling out the contribution of *rpoE_C_-chrR-chrA* operon to H_2_O_2_ tolerance.

### 3.4 *rpoEc-chrR-chrA* operon contributes to CAZ susceptibility

**Figure 4:**
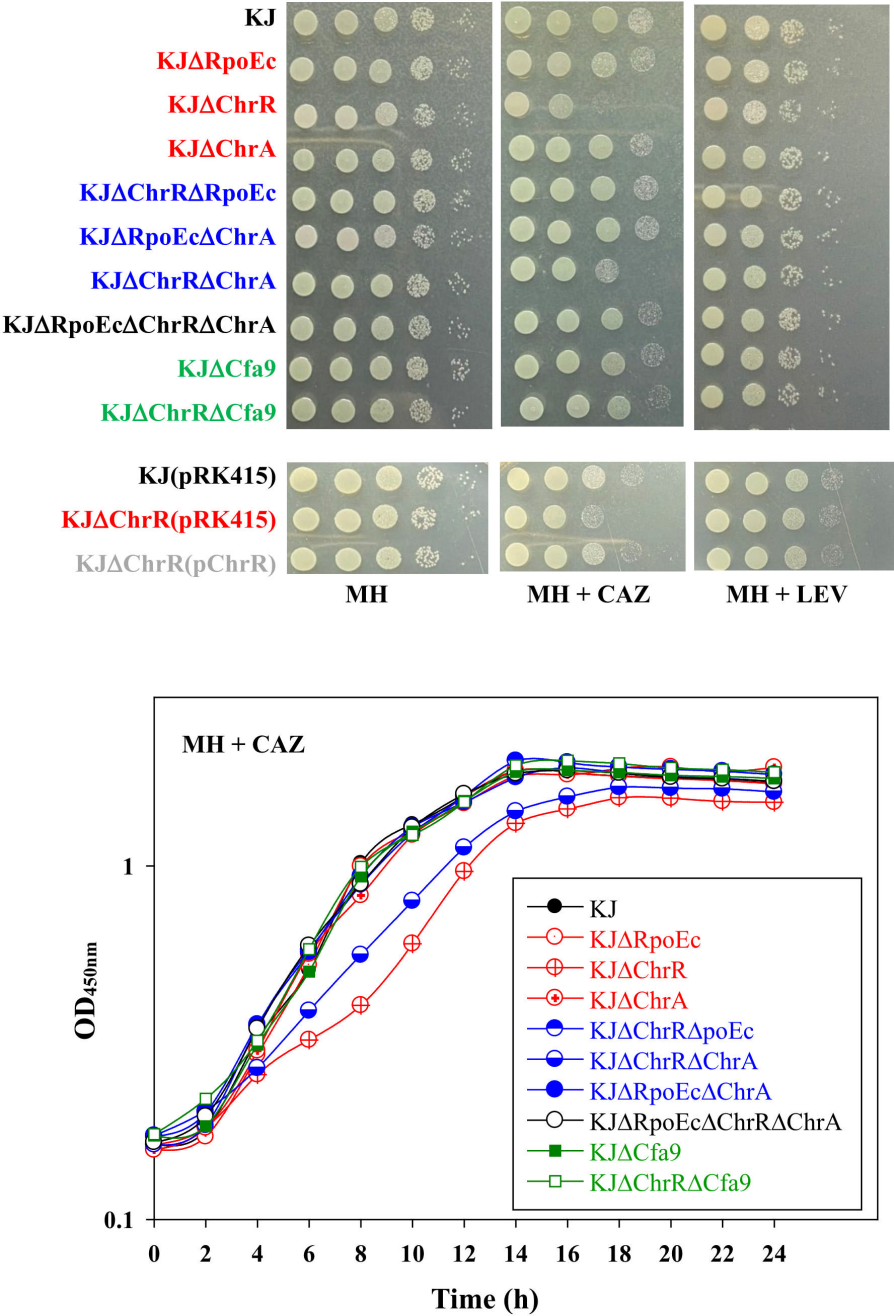
Role of *rpoEc-chrR-chrA* operon in antibiotic susceptibility. For cell viability, the logarithmic-phase bacterial cells (2 × 10^5^ CFU/μL) were 10-fold serially diluted. Bacterial aliquot (5 μL) was spotted onto MH agars without and with 120 μg/mL CAZ or 0.25 μg/mL LEV. After a 24-h incubation at 37°C, the growth of bacterial cells was observed. For growth curve, overnight culture was inoculated into MH broth with 200 μg/mL CAZ at an initial OD_450_ of 0.15. Bacterial growth was monitored for 24 h. The image and graph are representatives of at least three replicated experiments.

*S. maltophilia* is intrinsically resistant to various antibiotics (Crossman et al., 2008). Clinically, CAZ and fluoroquinolones are the choices for the treatment of *S. maltophilia* infection. Stress alleviation systems can cross-protect bacteria from antibiotics (Shin et al., 2020). Therefore, we assessed the involvement of *rpoEc-chrR-chrA* operon in susceptibility to CAZ and levofloxacin (LEV) susceptibility. The viabilities of wild-type KJ and its derived mutants in MH agar or broth containing CAZ and LEV were assessed. Of the three single-deletion mutants, KJΔChrR showed compromised viability in a medium containing CAZ, and viability was restored by ChrR complementation (**Figure 4**). Furthermore, KJΔChrRΔRpoEc and KJΔChrRΔChrA displayed viability almost comparable to wild-type KJ (**Figure 4**). Collectively, *ΔchrR*-mediated *rpoEc* and *chrA* upregulation contributed to an increase in CAZ susceptibility. With respect to LEV susceptibility, all *rpoEc-chrR-chrA* operon-associated mutants displayed viability comparable to wild-type KJ (**Figure 4**), tentatively ruling out the involvement of *rpoEc-chrR-chrA* operon in LEV susceptibility.

The mechanisms responsible for CAZ susceptibility in *S. maltophilia* can be attributed to β-lactamase-mediated and non-β-lactamase-mediated mechanisms (Huang et al., 2021). To assess whether β-lactamase is involved in the *ΔchrR*-mediated CAZ susceptibility increase, CAZ-induced β-lactamase activities of KJ, KJΔChrR, KJΔChrRΔRpoEc, and KJΔChrRΔChrA were determined. All mutants tested displayed comparable CAZ-induced β-lactamase activities with wild-type KJ (**Supplementary Figure S1**), indicating that σ^Ec^ activation-mediated increase of β-lactam susceptibility is irrelated to β-lactamase activity.

### 3.5 Regulation of *rpoEc-chrR-chrA* operon expression

**Figure 5:**
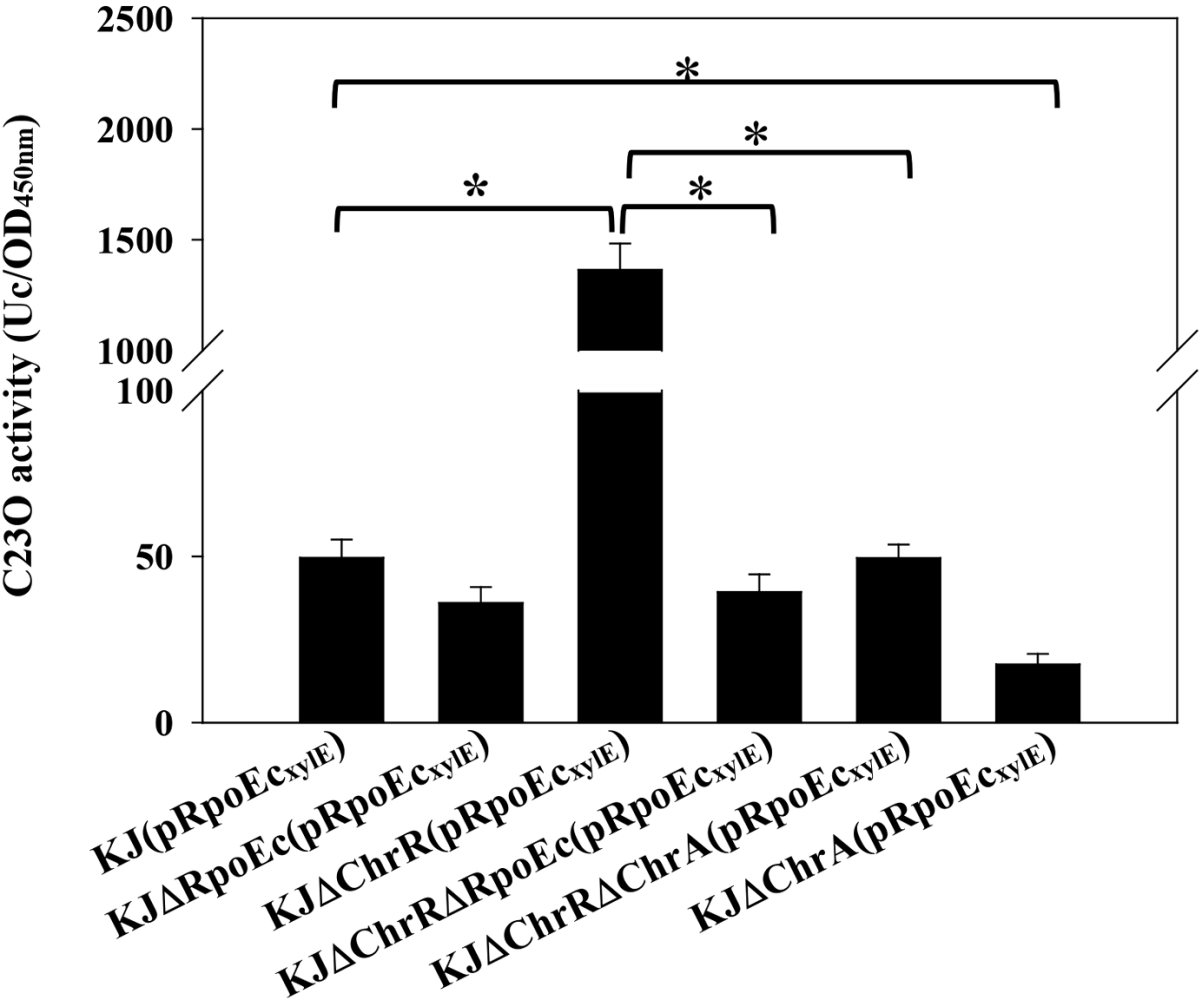
Regulation of *rpoEc-chrR-chrA* operon expression. Overnight cultures of bacterial cells tested were subcultured into fresh LB broth with an initial OD_450_ of 0.15. After 5-h culture, the C23O activity was measured. One unit of C23O activity (Uc) was defined as the amount of C23O that converted 1 nmol of catechol per min. The C23O specific activity was expressed as Uc/OD_450_. Bars represent the average values from three independent experiments. Error bars indicate the standard deviations for three triplicate samples. A two-sided Welch’s *t*-test and Bonferroni’s correction were used to determine statistical significance. *P <0.01.

We constructed a *P_rpoEc_* and *xylE* transcriptional fusion construct, pRpoEc_xylE_ (**Supplementary Table S1**), to assess the expression of *rpoEc-chrR-chrA* operon by C23O determination. Plasmid pRpoEc_xylE_ was transformed into the wild-type and its derived *rpoEc-chrR-chrA* operon*-*associated mutants (**Supplementary Table S1**) to assess the autoregulation circuit. KJ(pRpoEc_xylE_) exhibited moderate C23O activity, supporting the intrinsic expression of *rpoEc-chrR-chrA* operon. Compared to that of wild-type KJ, the promoter activity of *rpoEc-chrR-chrA* operon was significantly increased in KJΔChrR and reverted to wild-type levels in KJΔChrRΔRpoEc and KJΔChrRΔChrA (**Figure 5**). These results are consistent with the previous understanding of σ^E^-ChrR system that ChrR functions as an anti-σ^E^ and σ^E^ imposes autoregulation on its own expression (Donohue, 2019). A more interesting finding is that ChrA played a positive role in *ΔchrR*-mediated *rpoEc* upregulation (**Figure 5**). To further clarify this, the promoter activity of *rpoEc-chrR-chrA* operon in KJΔChrA was assessed. KJΔChrA(pRpoEc_xylE_) displayed lower C23O activity than KJ(pRpoEc_xylE_) (**Figure 5**), supporting that ChrA plays a positive role in regulating the transcriptional expression of *rpoEc-chrR-chrA* operon.

Given the contribution of *rpoEc-chrR-chrA* operon to MD tolerance (**Figure 3A**) and CAZ susceptibility (**Figure 4**), we wondered whether MD and CAZ were the stimuli that induced the expression of *rpoEc-chrR-chrA* operon. The C23O activity expressed by KJ(pRpoEc_xylE_) under MD and CAZ challenges was determined. MD- and CAZ-treated KJ(pRpoEc_xylE_) exhibited C23O activity comparable to the untreated counterpart (**Supplementary Figure S2**), indicating that MD and CAZ are not the stimuli that activate σ^Ec^.

### 3.6 *smlt2375-smlt2367* genes upregulation contributes to σ^Ec^ activation-mediated increase in MD tolerance and CAZ susceptibility

**Figure 6:**
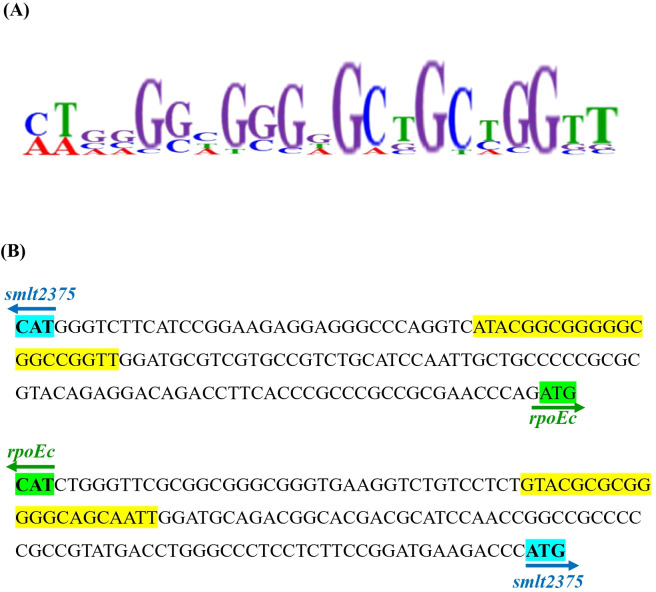
In *silico* analysis of putative σ^Ec^ box of *S. maltophilia* KJ. **(A)** Sequence logo of the putative σ^Ec^ box of *S. maltophilia*. Identification of putative σ^Ec^ box was performed using HEME program. The inputs included the 500-base region upstream from the 24 DEGs revealed by transcriptome analysis of KJ and KJΔChrR. **(B)** The intergenic region of *rpoEc* and *smlt2375*, and the putative σ^Ec^ box. *rpoEc* and *smlt2375* code σ^Ec^ and a hypothetical protein, respectively. The start codon for *smlt2375* and *rpoEc* are highlighted in blue and green, respectively. The putative σ^Ec^ box of *rpoEc* and *smlt2375* are highlighted in yellow.

To elucidate the mechanism underlying the σ^Ec^ activation-mediated increase in MD tolerance and CAZ susceptibility, RNA-seq transcriptome analysis of wild-type KJ, KJΔChrR, and KJΔChrRΔRpoEc was performed once. Differentially expressed genes (DEGs) were considered significant if the change in transcripts per kilobase million (TPM) between KJ and KJΔChrR was greater than three-fold. Twenty-four DEGs were revealed (**Supplementary Table S2**). Of them, 23 genes were upregulated and one was downregulated in KJΔChrR (**Supplementary Table S2**). Furthermore, the TPM values of the 24 DEGs in KJΔChrRΔRpoEc were significantly reverted (**Supplementary Table S2**), indicating that these 24 genes were members of the ChrR-σ^E^ regulatory circuit. To validate the transcriptome data, we performed qRT-PCR to probe the genes *rpoEc*, *smlt2373*, *smlt2382*, *cytB*, *L1*, and *L2*. The results supported the reliability of the transcriptome results (**Supplementary Figure S3**). By inputting the 500-base region upstream of the 24 DEGs into the motif search program MEME (Bailey et al., 2009), we generated a putative consensus DNA-binding motif of σ^Ec^ (σ^Ec^ box) (**Figure 6A**). **Figure 6B** shows the putative σ^Ec^ box located upstream of *rpoEc a*nd *smlt2375*.

As β-lactam resistance of *S. maltophilia* is strongly linked to PG homeostasis and β-lactamase induction (Huang et al., 2021), we checked the TPM values of L1, L2, and 37 known PG homeostasis-associated genes. No significant DEGs were identified among the 39 genes (**Supplementary Table S3**). β-lactamase activity and transcriptome analyses (**Supplementary Figure S1**, **Supplementary Table S3**) suggested the involvement of a non-β-lactamase mechanism in the σ^Ec^ activation-mediated increase in β-lactam susceptibility.

Among the identified DEGs (**Supplementary Table S2**), a nine-gene cluster, *smlt2375-smlt2367*, was the most significantly upregulated (**Table 1**). The nine genes were located upstream of *rpoEc-chrR-chrA* operon and divergently transcribed (**Figure 1**). Furthermore, these genes were simultaneously upregulated in KJΔChrR but significantly reverted in KJΔChrRΔRpoEc (**Table 1**). These observations strongly suggest that these nine genes form an operon regulated by σ^Ec^. The annotations and locations of the proteins encoded by *smlt2375-smlt2367* cluster are summarized in **Table 1**. The nine-gene cluster appeared to be involved in membrane lipid modification as it encoded a fatty acid desaturase (Smlt2374), an oxidoreductase (Smlt2373), two cyclopropane-fatty-acyl phospholipid synthase (CFA synthases) (Smlt2371 and Smlt2368), and five hypothetical proteins (**Table 1**). To investigate the role of the nine-gene upregulation in *ΔchrR*-mediated increase in MD tolerance and CAZ susceptibility, a nine-gene deletion mutant was constructed in wild-type KJ and KJΔChrR, yielding KJΔCfa9 and KJΔChrRΔCfa9, respectively (**Supplementary Table S1**). KJΔCfa9 and wild-type KJ showed comparable MD tolerance and CAZ susceptibility; however, compared to those in KJΔChrR, MD tolerance and CAZ susceptibility were almost reverted to wild-type levels in KJΔChrRΔCfa9 (**Figures 3A**, **4**), indicating that *smlt2375-smlt2367* genes upregulation contributes to *ΔchrR*-mediated increase in MD tolerance and CAZ susceptibility.

**Table 1:** Transcripts Per Kilobase Million (TPM) values of selected genes in KJ, KJΔChrR, and KJΔChrRΔRpoEc, revealed by transcriptome analysis.

Smlt	Protein		TPM^a^ (Fold change relative to KJ^b^)		
	Annotation	Location	KJ	ΔChrR	ΔChrRΔRpoEc
2367	lipocalin family protein	periplasm	17.33 (1)	2867.37 (+165.47)	7.20 (-2.40)
2368	CfaS1, cyclopropane-fatty-acyl-phospholipid synthase	cytosol	16.22 (1)	3352.29 (+206.67)	3.37 (-4.81)
2369	hypothetical protein	inner membrane	2.93 (1)	576.64 (+196.86)	1.00 (-2.93)
2370	hypothetical protein	inner membrane	2.55 (1)	532.77 (+208.58)	0.80 (-3.18)
2371	CfaS2, cyclopropane-fatty-acyl-phospholipid synthase	cytosol	4.81 (1)	1413.76 (+293.67)	1.13 (-4.25)
2372	hypothetical protein	cytosol	5.48 (1)	1459.26 (+266.45)	0.81 (-6.76)
2373	FAD-dependent oxidoreductase	cytosol	4.90 (1)	1535.77 (+313.29)	0.74 (-6.62)
2374	acyl-CoA desaturase	inner membrane	4.12 (1)	2088.48 (+506.88)	2.13 (-1.93)
2375	hypothetical protein	periplasm	1.07 (1)	743.80 (+697.41)	0.78 (-1.37)
2377	σ^Ec^	cytosol	35.09 (1)	1587.08 (+45.23)	17.94 (-1.95)
2378	ChrR	inner membrane	24.66 (1)	800.91 (+32.47)	9.56 (-2.57)
2379	ChrA, Oxidoreductase	cytosol	49.87 (1)	940.68 (+18.86)	26.16 (-1.90)

a TPM, Transcripts Per Kilobase Million.

b Negative fold changes represent genes that were significantly downregulated compared to wild-type KJ, whereas positive fold changes represent upregulation compared to wild-type KJ.

## 4 Discussion

σ^E^-ChrR system was first reported in photosynthetic microorganisms, such as *R. sphaeroides*, due to its role in the alleviation of stress induced by singlet oxygen, which is an inevitable by-product of photosynthesis (Anthony et al., 2004). Later, a core σ^E^-ChrR regulon was discovered in non-photosynthetic α- and γ-proteobacteria, such as *Azospirillum brasilense*, *Caulobacter crescentus*, *Shewanella oneidensis*, *Pseudomonas syringae* pv. tomato, and *Vibrio cholerae* (Gupta et al., 2013; Lourenço and Gomes, 2009; Dai et al., 2015; Butcher et al., 2017; Tardu et al., 2017). These known σ^E^-ChrR regulons are involved in the alleviation of singlet oxygen stress. In this study, we revealed that *rpoEc-chrR-chrA* operon was not involved in singlet oxygen stress alleviation but contributed to superoxide tolerance and CAZ susceptibility in *S. maltophilia* (**Figures 2**, **3A**, **4**). A similar function was shown earlier in *A. brasilense* Sp245, in which the *rpoE2-chrR2* system is involved in superoxide and antibiotic tolerance (Gupta et al., 2013). Sigma E2 activation in *A. brasilens* Sp245 confers resistance to β-lactam and nalidixic acid (Gupta et al., 2013); however, σ^Ec^ activation increases the β-lactam susceptibility of *S. maltophilia* (**Figure 4**).

The *rpoE-chrR* system is an effector of singlet oxygen stress, and both genes are generally organized into an operon in bacteria. Unlike the two-gene *rpoE-chrR* operon, *rpoEc* and *chrR* of *S. maltophilia* are located in the three-gene *rpoEc-chrR-chrA* operon (**Figure 1A**). Similarly, a three-gene *rpoE-chrR-VC2303* operon is observed in *V. cholerae* (Tardu et al., 2017). The protein encoded by *VC2303* is annotated as a hypothetical protein and its significance in the RpoE-ChrR system has not characterized. In this study, we verified that ChrA of *S. maltophilia* plays a positive role in the expression of *rpoEc* regulon (**Figure 5**). To consider the underlying mechanism responsible for ChrA function, the *rsbV-rsbW-sigB-rsbX* operon of *Bacillus subtilis* provides some ideas. *sigB*, *rsbW*, and *rsbV* encode the sigma factor σ^B^, anti-σ^B^ (RsbW), and anti-anti-σ^B^ (RsbV), respectively (Kazmierczak et al., 2005). Similar to most ECF σ factors, σ^B^ autoregulates the *rsbV-rsbW-sigB-rsbX* operon expression. RsbX plays a negative role in σ^B^ activity through its phosphatase activity (Voelker et al., 1997). Here, inactivation of *chrA* decreased the expression of *rpoEc-chrR-chrA* operon (**Figure 5**). As ChrA is predicted to be a cytoplasmic protein with an NAD(P)-binding domain that functions as an oxidoreductase, we speculated that ChrA plays a positive role in modulating σ^Ec^ activity and this modulation may be oxidoreductase activity involved.

Transcriptome analysis revealed the genes regulated by *rpoEc-chrR* pair. Among the DEGs (**Supplementary Table S2**), three upregulated gene clusters and a downregulated gene attracted our attention, that is, *smlt2375-smlt2367*, *smlt2382-smlt2380*, *yceA-cybB-yceB* operon (*smlt3627-smlt3629*), and *smlt0227*. *smlt2375-smlt2367* gene cluster was the most upregulated in KJΔChrR. Because of the presence of a fatty acid desaturase gene (*smlt2374*) and two CFA synthase genes (*smlt2371* and *smlt2368*), *smlt2375*-*smlt2367* gene cluster may be involved in membrane lipid modification. CFA synthase catalyzes the modification of the acyl chains of membrane phospholipids through the methylation of unsaturated fatty acyl chains to generate cyclopropane-fatty-acyl-phospholipid (CFA) (Grogan and Cronan, 1997). Modification of membrane lipids by CFA synthase can alter the biophysical properties of membranes and protect cells against drastic environmental perturbations such as oxidative stress, high ionic strength, and acid stress (Grogan and Cronan, 1986, 1997; Tardu et al., 2017; Chang and Cronan, 1999; Jiang et al., 2019). Membrane lipid modification also modulates membrane permeability and fluidity, thereby altering bacterial susceptibility to antibiotics and antimicrobial peptides (Schmidt et al., 2018). Integrating our finding that β-lactamase activity is not the critical factor responsible for σ^Ec^ activation-mediated increase of β-lactam susceptibility. Therefore, we speculated that the upregulation of *smlt2375-smlt2367* cluster in KJΔChrR may alter membrane properties against MD-mediated oxidative stress and increase membrane permeability to CAZ, which increases MD tolerance and CAZ susceptibility. *smlt2382-smlt2380* gene cluster is located downstream of *rpoEc-chrR-chrA* operon and divergently transcribed. The proteins encoded by *smlt2382-smlt2380* are the members of a two-component regulatory system, including sensor kinase, response regulator, and hybrid sensor kinase/response regulator, respectively. Thus, a regulatory interconnect between *rpoEc-chrR-chrA* operon and *smlt2382-smlt2380* gene cluster highly exists. Furthermore, *yceA-cybB-yceB* operon is the member of *rpoEc* regulon. The contribution of *yceA-cybB-yceB* operon to MD tolerance has been reported recently (Liao et al., 2023). Therefore, the *smlt2375-smlt2367* cluster upregulation may not be the sole factor leading to MD tolerance increase in KJΔChrR. The contribution of *yceA-cybB-yceB* operon upregulation cannot be ignored. *smlt0227* is the sole downregulated DEG in KJΔChrR. The protein encoded by *smlt0227* is annotated as a major facilitator superfamily (MFS) transporter. MFS transporter plays a crucial role in a multitude of physiological processes and its involvement in stress tolerance and antibiotic susceptibility has been widely reported (Pao et al., 1998; Nag and Mehra, 2021).

**Figure 7:**
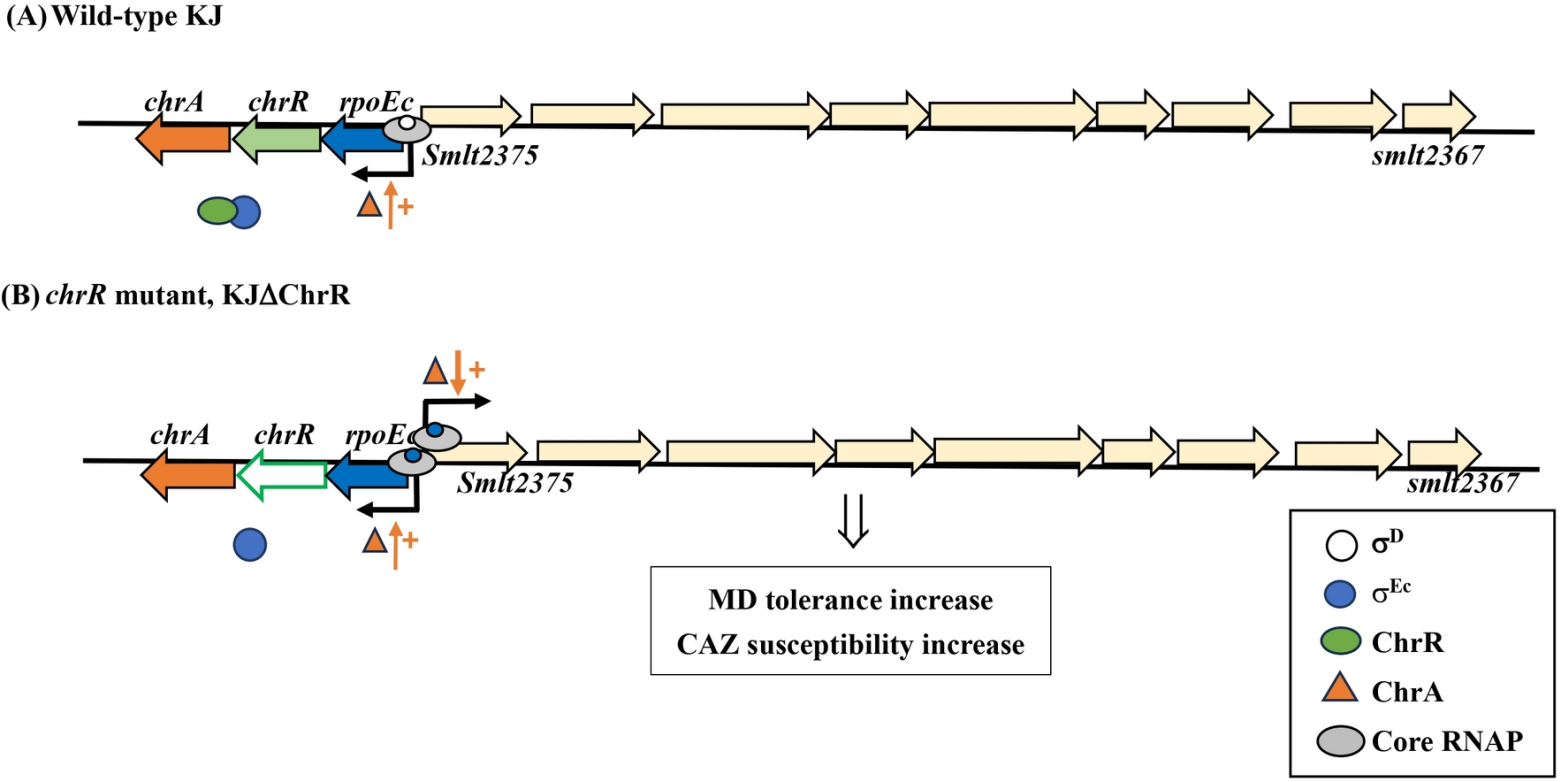
The proposed model for σ^Ec^ activation-mediated increase in MD tolerance and CAZ susceptibility. In the wild-type KJ, *rpoEc-chrR-chrA* operon is moderately expressed via σ^D^-driven RNA polymerase. The σ^Ec^ protein is sequestered by ChrR **(A)**. In the *chrR* mutant, the free form σ^Ec^ and core RNA polymerase (RNAP) form a holoenzyme, which drives the expression of σ^Ec^ regulon, including *rpoEc-chrR-chrA* operon and *smlt2375-smlt2367* cluster. ChrA protein plays a positive role in the transcriptional-level expression of σ^E^ regulon. The upregulation of *smlt2375-smlt2367* cluster contributes to the σ^Ec^ activation-mediated increase in MD tolerance and CAZ susceptibility **(B)**.

Promoter activity and transcriptome assay revealed that *rpoEc-chrR-chrA* operon exhibited moderate expression, indicating that *rpoEc-chrR-chrA* operon was transcribed by σ^D^-driven RNAP in logarithmically grown KJ cells. Under these conditions, translated σ^Ec^ was sequestered via ChrR binding (**Figure 7A**). In the presence of stimuli, σ^Ec^ was released from ChrR and activated the expression of σ^Ec^ regulon. In this study, KJΔChrR mimicked the concept of σ^Ec^ activation. A positive autoregulation was observed in the *rpoEc-chrR-chrA* operon and ChrA played a positive role in this regulatory circuit. Furthermore, activated σ^Ec^ upregulated the expression of *smlt2375-smlt2367* gene cluster, thus increasing the MD tolerance and CAZ susceptibility (**Figure 7B**).

## Data Availability

The datasets presented in this study can be found in online repositories. The names of the repository/repositories and accession number(s) can be found in the article/**Supplementary Material**.

## References

[B1] Anthony J. R. Newman J. D. Donohue T. J. (2004). Interactions between the *Rhodobacter sphaeroides* ECF sigma factor, *σE*, and its anti-sigma factor, ChrR. J. Mol. Biol. 341, 345–360. doi: 10.1016/j.jmb.2004.06.018 15276828 PMC2796631

[B2] Bailey T. L. Boden M. Buske F. A. Frith M. Grant C. E. Clementi L.. (2009). MEME suite: tools for motif discovery and searching. Nucleic Acids Res. 37, W202–W208. doi: 10.1093/nar/gkp335 19458158 PMC2703892

[B3] Brahmachari G. Karmakar I. (2020). Visible light-induced and singlet oxygen-mediated photochemical conversion of 4-hydroxy-α-benzopyrones to 2-hydroxy-3-oxo-2,3-dihydrobenzofuran-2-carboxamides/carboxylates using rose Bengal as a photosensitizer. J. Org. Chem. 85, 8851–8864. doi: 10.1021/acs.joc.0c00726 32543197

[B4] Butcher B. G. Bao Z. Wilson J. Stodghill P. Swingle B. Filiatrault M.. (2017). The ECF sigma factor, PSPTO_1043, in *Pseudomonas syringae* pv. tomato DC3000 is induced by oxidative stress and regulates genes involved in oxidative stress response. PloS One 12, e0180340. doi: 10.1371/journal.pone.0180340 28700608 PMC5507510

[B5] Chang Y. Y. Cronan J. E. Jr (1999). Membrane cyclopropane fatty acid content is a major factor in acid resistance of *Escherichia coli* . Mol. Microbiol. 33, 249–259. doi: 10.1046/j.1365-2958.1999.01456.x 10411742

[B6] Chen C. H. Huang C. C. Chung T. C. Hu R. M. Huang Y. W. Yang T. C. (2011). Contribution of resistance-nodulation-division efflux pump operon *smeU1-V-W-U2-X* to multidrug resistance of *Stenotrophomonas maltophilia* . Antimicrob. Agents Chemother. 55, 5826–5833. doi: 10.1128/AAC.00317-11 21930878 PMC3232770

[B7] Crossman L. C. Gould V. C. Dow J. M. Vernikos G. S. Okazaki A. Sebaihia M.. (2008). The complete genome, comparative and functional analysis of *Stenotrophomonas maltophilia* reveals an organism heavily shielded by drug resistance determinants. Genome Biol. 9, R74. doi: 10.1186/gb-2008-9-4-r74 18419807 PMC2643945

[B8] Dai J. Wei H. Tian C. Damron F. H. Zhou J. Qiu D. (2015). An extracytoplasmic function sigma factor-dependent periplasmic glutathione peroxidase is involved in oxidative stress response of. Shewanella oneidensis. BMC Microbiol. 15, 34. doi: 10.1186/s12866-015-0357-0 25887418 PMC4336711

[B9] Davis M. C. Kesthely C. A. Franklin E. A. MacLellan S. R. (2017). The essential activities of the bacterial sigma factor. Can. J. Microbiol. 63, 89–99. doi: 10.1139/cjm-2016-0576 28117604

[B10] DeRosa M. C. Crutchley R. J. (2002). Photosensitized singlet oxygen and its applications. Coord Chem. Rev. 223-224, 351–371. doi: 10.1016/S0010-8545(02)00034-6

[B11] Di Mascio P. Briviba K. Sasaki S. T. Catalani L. H. Medeiros M. H. Bechara E. J.. (1997). The reaction of peroxynitrite with tert-butyl hydroperoxide products singlet molecular oxygen. Biol. Chem. 378, 1071–1074. 9348119

[B12] Donohue T. J. (2019). Shedding light on a Group IV (ECF11) alternative σ factor. Mol. Microbiol. 112, 374–384. doi: 10.1111/mmi.v112.2 31111523 PMC6852236

[B13] Glaeser J. Nuss A. M. Berghoff B. A. Klug G. (2011). Singlet oxygen stress in microorganisms. Adv. Microb. Physiol. 58, 141–173. doi: 10.1016/B978-0-12-381043-4.00004-0 21722793

[B14] Grogan D. W. Cronan J. E. (1986). Characterization of *Escherichia coli* mutants completely defective in synthesis of cyclopropane fatty-acids. J. Bacteriol 166, 872–877. doi: 10.1128/jb.166.3.872-877.1986 3519583 PMC215207

[B15] Grogan D. W. Cronan J. E. Jr. (1997). Cyclopropane ring formation in membrane lipids of bacteria. Microbiol. Mol. Biol. Rev. 61, 429–441. doi: 10.1128/mmbr.61.4.429-441 9409147 PMC232619

[B16] Gruber T. M. Gross C. A. (2003). Multiple sigma subunits and the partitioning of bacterial transcription space. Annu. Rev. Microbiol. 57, 441–466. doi: 10.1146/annurev.micro.57.030502.090913 14527287

[B17] Gupta N. Kumar S. Mishra M. N. Tripathi A. K. (2013). A constitutively expressed pair of *rpoE2-chrR2* in *Azospirillum brasilense* Sp7 is required for survival under antibiotic and oxidative stress. Microbiol. (Reading) 159, 205–218. doi: 10.1099/mic.0.061937-0 23059974

[B18] Helmann J. D. (2002). The extracytoplasmic function (ECF) sigma factors. Adv. Microb. Physiol. 46, 47–110. doi: 10.1016/S0065-2911(02)46002-X 12073657

[B19] Ho T. D. Ellermeier C. D. (2012). Extra cytoplasmic function σ factor activation. Curr. Opin. Microbiol. 15, 182–188. doi: 10.1016/j.mib.2012.01.001 22381678 PMC3320685

[B20] Huang Y. W. Huang H. H. Huang K. H. Chen W. C. Lin Y. T. Hsu C. C.. (2019). AmpI function as an iron exporter to alleviate -lactam-mediated reactive oxygen species stress in Stenotrophomonas maltophilia. Antimicrob. Agents Chemother. 63, e02467–e02418. doi: 10.1128/AAC.02467-18 30745379 PMC6437473

[B21] Huang Y. W. Liou R. S. Lin Y. T. Huang H. H. Yang T. C. (2014). A linkage between SmeIJK efflux pump, cell envelope integrity, and σ^E^-mediated envelope stress response in *Stenotrophomonas maltophilia* . PloS One 9, e111784. doi: 10.1371/journal.pone.0111784 25390933 PMC4229105

[B22] Huang H. H. Wu B. K. Li L. H. Lin Y. T. Yang T. C. (2021). Role of the PhoPQ two-component regulatory system in the β-lactam resistance of *Stenotrophomonas maltophilia* . J. Antimicrob. Chemother. 76, 1480–1486. doi: 10.1093/jac/dkab059 33739413

[B23] Jacoby G. A. (2009). AmpC beta-lactamases. Clin. Microbiol. Rev. 22, 161–182. doi: 10.1128/CMR.00036-08 19136439 PMC2620637

[B24] Jair H. W. Lu H. F. Huang Y. W. Pan S. Y. Lin I. L. Huang H. H.. (2019). Roles of the two-MnSOD system of *Stenotrophomonas maltophilia* in the alleviation of superoxide stress. Int. J. Mol. Sci. 20, 1770. doi: 10.3390/ijms20071770 30974814 PMC6479884

[B25] Jiang X. J. Duan Y. Zhou B. Guo Q. Wang H. Hang X.. (2019). The cyclopropane fatty acid synthase mediates antibiotic resistance and gastric colonization of *Helicobacter pylori* . J. Bacteriol 201, e00374–e00319. doi: 10.1128/JB.00374-19 PMC6755722 31358615

[B26] Kazmierczak M. J. Wiedmann M. Boor K. J. (2005). Alternative sigma factors and their roles in bacterial virulence. Microbiol. Mol. Biol. Rev. 69, 527–543. doi: 10.1128/MMBR.69.4.527-543.2005 16339734 PMC1306804

[B27] Li L. H. Shih Y. L. Huang J. Y. Wu C. J. Huang Y. W. Huang H. H.. (2020a). Protection from hydrogen peroxide stress relies mainly on AhpCF and KatA2 in Stenotrophomonas maltophilia. J. BioMed. Sci. 27, 37. doi: 10.1186/s12929-020-00631-4 32093695 PMC7041247

[B28] Li L. H. Wu C. M. Lin Y. T. Pan S. Y. Yang T. C. (2020b). Roles of FadRACB system in formaldehyde detoxification, oxidative stress alleviation, and antibiotic susceptibility in Stenotrophomonas maltophilia. J. Antimicrob. Chemother. 75, 2101–2109. doi: 10.1093/jac/dkaa173 32407477

[B29] Liao C. H. Ku R. H. Li L. H. Wu C. M. Yang T. C. (2023). Role of *yceA-cybB-yceB* operon in oxidative stress tolerance, swimming motility and antibiotic susceptibility of *Stenotrophomonas maltophilia* . J. Antimicrob. Chemother. 78, 1891–1899. doi: 10.1093/jac/dkad179 37283194

[B30] Liao C. H. Lu H. F. Huang H. H. Chen Y. Li L. H. Lin Y. T.. (2022). The *fciTABC* and *feoABI* systems contribute to ferric citrate acquisition in *Stenotrophomonas maltophilia* . J. BioMed. Sci. 29, 26. doi: 10.1186/s12929-022-00809-y 35477574 PMC9047314

[B31] Lin Y. T. Huang Y. W. Liou R. S. Chang Y. C. Yang T. C. (2014). MacABCsm, an ABC-type tripartite efflux pump of *Stenotrophomonas maltophilia* involved in drug resistance, oxidative and envelope stress tolerance and biofilm formation. J. Antimicrob. Chemother. 69, 3221–3226. doi: 10.1093/jac/dku317 25139838

[B32] Livak K. J. Schmittgen T. D. (2001). Analysis of relative gene expression data using real-time quantitative PCR and the 2(-Delta Delta C(T)) Method. Methods 25, 402–408. doi: 10.1006/meth.2001.1262 11846609

[B33] Lourenço R. F. Gomes S. L. (2009). The transcriptional response to cadmium, organic hydroperoxide, singlet oxygen and UV-A mediated by the σ^E^-ChrR system in Caulobacter crescentus. Mol. Microbiol. 72, 1159–1170. doi: 10.1111/j.1365-2958.2009.06714.x 19400803

[B34] Marles-Wright J. Lewis R. J. (2007). Stress responses of bacteria. Curr. Opin. Struct. Biol. 17, 755–760. doi: 10.1016/j.sbi.2007.08.004 17920859

[B35] Mascher T. (2023). Past, present, and future of extracytoplasmic function σ factors: distribution and regulatory diversity of the third pillar of bacterial signal transduction. Annu. Rev. Microbiol. 77, 625–644. doi: 10.1146/annurev-micro-032221-024032 37437215

[B36] Mojica M. F. Humphries R. Lipuma J. J. Mathers A. J. Rao G. G. Shelburne S. A.. (2022). Clinical challenges treating *Stenotrophomonas maltophilia* infections: an update. JAC Antimicrob. Resist. 4, dlac040. doi: 10.1093/jacamr/dlac040 35529051 PMC9071536

[B37] Nag A. Mehra S. (2021). A major facilitator superfamily (MFS) efflux pump, SCO4121, from *Streptomyces coelicolor* with roles in multidrug resistance and oxidative stress tolerance and its regulation by a MarR regulator. Appl. Environ. Microbiol. 87, e02238–e02220. doi: 10.1128/AEM.02238-20 33483304 PMC8091613

[B38] Paget M. S. (2015). Bacterial sigma factors and anti-sigma factors: structure, function and distribution. Biomolecules 5, 1245–1265. doi: 10.3390/biom5031245 26131973 PMC4598750

[B39] Pan S. Y. Shih Y. L. Huang H. H. Li L. H. Lin Y. T. Yang T. C. (2022). The involvement of PacIRA system of *Stenotrophomonas maltophilia* in the uptake of *Pseudomonas aeruginosa* pyochelin and intraspecies competition for iron acquisition. J. Microbiol. Immunol. Infect. 55, 273–281. doi: 10.1016/j.jmii.2021.03.001 33811013

[B40] Pao S. S. Paulsen I. T. Saier M. H. Jr. (1998). Major facilitator superfamily. Microbiol. Mol. Biol. Rev. 62, 1–34. doi: 10.1128/MMBR.62.1.1-34.1998 9529885 PMC98904

[B41] Sánchez M. B. (2015). Antibiotic resistance in the opportunistic pathogen *Stenotrophomonas maltophilia* . Front. Microbiol. 6, 658. doi: 10.3389/fmicb.2015.00658 26175724 PMC4485184

[B42] Schmidt R. Yonghong D. Hoffmann R. (2018). Phospholipid composition of the outer membrane of *Escherichia coli* influences its susceptibility against antimicrobial peptide apidaecin 1b. Diagn. Microbiol. Infect. Dis. 90, 316–323. doi: 10.1016/j.diagmicrobio.2017.11.008 29329756

[B43] Sharma P. Jha A. B. Dubey R. S. Pessarakli M. (2012). Reactive oxygen species, oxidative damage, and antioxidative defense mechanism in plants under stressful conditions. J. Bot. 2012, 1–26. doi: 10.1155/2012/217037

[B44] Shih Y. L. Wu C. M. Lu H. F. Li L. H. Lin Y. T. Yang T. C. (2022). Involvement of the *hemP-hemA-smlt0796-smlt0797* operon in hemin acquisition by *Stenotrophomonas maltophilia* . Microbiol. Spectr. 10, e0032122. doi: 10.1128/spectrum.00321-22 35658602 PMC9241770

[B45] Shin B. Park C. Park W. (2020). Stress responses linked to antimicrobial resistance in *Acinetobacter* species. Appl. Microbiol. Biotechnol. 104, 1423–1435. doi: 10.1007/s00253-019-10317-z 31900550

[B46] Sun S. Zhou J. (2018). Molecular mechanisms underlying stress response and adaptation. Thorac. Cancer 9, 218–227. doi: 10.1111/tca.2018.9.issue-2 29278299 PMC5792716

[B47] Tardu M. Bulut S. Kavakli I. H. (2017). MerR and ChrR mediate blue light induced photo-oxidative stress response at the transcriptional level in *Vibrio cholera* . Sci. Rep. 7, 40817. doi: 10.1038/srep40817 28098242 PMC5241685

[B48] Triantaphylidès C. Havaux M. (2009). Singlet oxygen in plants: production, detoxification and signaling. Trends Plant Sci. 14, 219–228. doi: 10.1016/j.tplants.2009.01.008 19303348

[B49] Voelker U. Luo T. Smirnova N. Haldenwang W. (1997). Stress activation of *Bacillus subtilis* sigma B can occur in the absence of sigma B negative regulator RsbX. J. Bacteriol 179, 1980–1984. doi: 10.1128/jb.179.6.1980-1984.1997 9068644 PMC178922

[B50] Wu C. J. Chen Y. Li L. H. Wu C. M. Lin Y. T. Ma C. H.. (2022). Roles of SmeYZ, SbiAB, and SmeDEF efflux systems in iron homeostasis of *Stenotrophomonas maltophilia* . Microbiol. Spectr. 10, e0244821. doi: 10.1128/spectrum.02448-21 35647692 PMC9241820

[B51] Wu C. J. Chiu T. T. Lin Y. T. Huang Y. W. Li L. H. Yang T. C. (2018). Role of *smeU1VWU2X* operon in alleviation of oxidative stresses and occurrence of sulfaethoxazole-trimethoprim-resistant mutants in *Stenotrophomonas maltophilia* . Antimicrob. Agents Chemother. 62, e02114–7. doi: 10.1128/AAC.02114-17 29203487 PMC5786817

[B52] Yang T. C. Huang Y. W. Hu R. M. Huang S. C. Lin Y. T. (2009). AmpDI is involved in expression of chromosomal L1 and L2 β-lactamases of *Stenotrophomonas maltophilia* . Antimicrob. Agents Chemother. 53, 2902–2907. doi: 10.1128/AAC.01513-08 19414581 PMC2704650

