# Supplementary material for: Roles of the rpoEc-chrR-chrA operon in superoxide tolerance and β-lactam susceptibility of Stenotrophomonas maltophilia
Source: Front Cell Infect Microbiol. 2025 Feb 4;15:1492008. doi: 10.3389/fcimb.2025.1492008 (PMC11832516; doi:10.3389/fcimb.2025.1492008)
Supplement: Supplementary file 1 [file DataSheet1.docx]

**Table S1 Bacterial strains, plasmids, and primers used in this study**

| Strain or plasmid | Genotype or properties | Reference |
| --- | --- | --- |
| ***S. maltophilia***  KJ  KJ∆RpoEc  KJ∆ChrR  KJΔChrA  KJΔChrRΔRpoEc  KJΔChrRΔChrA  KJΔRpoEcΔChrA  KJΔRpoEcΔChrRΔChrA  KJΔCfa9  KJΔChrRΔCfa9 | A clinical *S. maltophilia* isolate  *S. maltophilia* KJ mutant of *rpoEc* gene; *ΔrpoEc*  *S. maltophilia* KJ mutant of *chrR* gene; *ΔchrR*  *S. maltophilia* KJ mutant of *chrA* gene; *ΔchrA*  *S. maltophilia* KJ mutant of *chrR* and *rpoEc* genes; *ΔchrR, ΔrpoEc*  *S. maltophilia* KJ mutant of *chrR* and *chrA* genes; *ΔchrR, ΔchrA*  *S. maltophilia* KJ mutant of *rpoEc* and *chrA* genes; *ΔrpoEc, ΔchrA*  *S. maltophilia* KJ mutant of *rpoEc-chrR-chrA* operon;  *ΔrpoEc, ΔchrR, ΔchrA*  *S. maltophilia* KJ mutant of *smlt2375-2367 cluster*; *Δsmlt2375-2367*  *S. maltophilia* KJ mutant of *chrR* and *smlt2375-2367 cluster*; *ΔchrR*, *Δsmlt2375-2367* | Hu et al., 2008  This study  This study  This study  This study  This study  ­  This study  This study  This study  This study |
| ***E. coli***  DH5α  S17-1 | F- φ80d/*acZΔM15* Δ(*lacZYA-argF*)*U169* *deoR recA1 endA1 hsdR17* (r_k_^-^ m_k_^+^) *phoA supE44λ* *thi-1 gyrA96 relA1*  λ*pir*^+^ mating strain | Invitrogen  Simon et al., 1986 |
| **Plasmids** |  |  |
| pEX18Tc | *sacB oriT*, Tc^r^ | Hoang et al., 1998 |
| pRK415 | Mobilizable broad-host-range plasmid cloning vector, RK2 origin; Tc^r^ | Keen et al., 1998 |
| pΔRpoEc  pΔChrR  p∆ChrA  pChrR  pRpoEc_xylE_  **Primers**  ChrA-C  RpoEcQ-F  RpoEcQ-R  ChrRQ-F  ChrRQ-R  ChrAQ-F  ChrAQ-R  RpoEcN-F  RpoEcN-R  RpoEcC-F  RpoEcC-R  ChrRC-F  ChrRC-R  ChrAC-F  ChrAC-R  2374Q82-F  2374Q82-R  2382Q105-F  2382Q105-R  CytBQ109-F  CytBQ109-R  L1Q73-F  L1Q73-R  L2Q92-F  L2Q92-R  16S rDNA-F  16S rDNA-R | pEX18Tc with an internal-deletion *rpoEc* gene; Tc^r^  pEX18Tc with an internal-deletion *chrR* gene; Tc^r^  pEX18Tc with an internal-deletion *chrA* gene; Tc^r^  pRK415 with intact *chrR* gene; Tc^r^  pRK415 with a 356-bp DNA fragment upstream from the *rpoEc* start codon and a *P_rpoEc_::xylE* transcriptional fusion, Tc^r^  GTAGAAGCAGGCATCGAACC  CTGGTGCGGATGTCCTATTT  CAAATGTGATTTGACCGTTCC  ATCTGGATGGAGACGTGGAG  AGCCAGCCACTGAACACC  CCTGTTGAAGCCCCTGTACC  GTGCAACCATCGCCTGAC  GTGGAATTCACGGTAGAGGAGGTCGC  GTCGGGCTCACCGGCCCAGTTCG  GGACCCGGGTGCAGGCAGCGGTGG  TGCTCTAGATGGGCACCGGCGAC  GCATCTAGACACCCGTACTTCGGC  CAGGATCCGTAATGCGCCCGTCCC  CGTGGTACCCTGTTGAAGCCCC  CCGGAATTCGCCGTCCTGTTCA  CATCACGGCGTTCTATCACC  GATGACTGCGAACACGAACT  AATCCAGCTTCCAGCATCAG  CAAGCACGTCCTTCTTTTCC  CCAGATCCTGCATTGGTTG  CGCGGTATAGACCCAGAAAT  GGTCACCTGCTGGACAACAT  CATGGCTGAGCAGGATCAAT  GATCTGCTCTCGCATGCAC  TCGCTGGTGATGATCGTG  GACCTTGCGCGATTGAATG  CGGATCGTCGCCTTGGT | This study  This study  This study  This study  This study  This study  This study  This study  This study  This study  This study  This study  This study  This study  This study  This study  This study  This study  This study  This study  This study  This study  This study  This study  This study  This study  This study  This study  This study  This study  Chen et al., 2011  Chen et al., 2011 |

Chen, C. H., Huang, C. C., Chung, T. C., Hu, R. M., Huang, Y. W., and Yang, T. C. (2011). Contribution of resistance-nodulation-division efflux pump operon *smeU1-V-W-U2-X* to multidrug resistance of *Stenotrophomonas maltophilia*. *Antimicrob Agents Chemother,* 55, 5826-5833.

Hoang, T. T., Karkhoff-Schweizer, R. R., Kutchma, A. J., and Schweizer, H. P. (1998). A broad-host-range Flp-FRT recombination system for site-specific excision of chromosomally-located DNA sequences: application for isolation of unmarked *Pseudomonas aeruginosa* mutants. *Gene*, 212, 77-86.

Hu, R. M., Huang, K. J., Wu, L. T., Hsiao, Y. J., and Yang, T. C. (2008). Induction of L1 and L2 beta-lactamases of *Stenotrophomonas maltophilia*. *Antimicrob Agents Chemother,* 52(3), 1198-1200.

Keen, N. T., Tamaki, S., Kobayashi, D., and Trollinger, D. (1998). Improved broad-host-range plasmids for DNA cloning in gram-negative bacteria. *Gene,* 70, 191-197.

Simon, R., O'Connell, M., Labes, M., and Puhler, A. (1986). Plasmid vector for the genetic analysis and manipulation of *Rhizobia* and other Gram-negative bacteria. *Methods Enzymol,* 118, 640-659.

**Table S2** **Differentially Expressed Genes (DEGs) of KJ, KJΔChrR, and KJΔChrRΔChrEc, revealed by transcriptome analysis**

| Smlt | TPM^a^ (Fold change relative to KJ^b^) | | | Protein |
| --- | --- | --- | --- | --- |
|  | **KJ** | **ΔChrR** | **ΔChrRΔRpoEc** |  |
| Upregulated in KJΔChrR | | | | |
| 0611 | 36.1 (1) | 759.3 (+21.0) | 33.6 (-1.07) | DNA modification/repair radical SAM protein |
| \| 2367 \| \| --- \| | 17.3 (1) | 2867.3 (+165.4) | 7.2 (-2.40) | lipocalin family protein |
| 2368 | 16.2 (1) | 3352.2 (+206.6) | 3.3 (-4.81) | CfaS, cyclopropane-fatty-acyl-phospholipid synthase |
| 2369 | 2.9 (1) | 576.6 (+196.8) | 1.0 (-2.93) | hypothetical protein |
| 2370 | 2.5 (1) | 532.7 (+208.5) | 0.8 (-3.18) | hypothetical protein |
| 2371 | 4.8 (1) | 1413.7 (+293.6) | 1.1 (-4.25) | CfaS, cyclopropane-fatty-acyl-phospholipid synthase |
| 2372 | 5.4 (1) | 1459.2 (+266.4) | 0.8 (-6.76) | hypothetical protein |
| 2373 | 4.9 (1) | 1535.7 (+313.2) | 0.7 (-6.62) | FAD-dependent oxidoreductase |
| 2374 | 4.1 (1) | 2088.4 (+506.8) | 2.1 (-1.93) | acyl-CoA desaturase |
| 2375 | 1.0 (1) | 743.8 (+697.4) | 0.7 (-1.37) | hypothetical protein |
| 2377 | 35.0 (1) | 1587.0 (+45.2) |  | σ^Ec^ |
| 2378 | 24.6 (1) |  |  | ChrR |
| 2379 | 49.8 (1) | 940.6 (+18.8) | 26.1 (-1.90) | ChrA, oxidoreductase |
| 2380 | 90.8 (1) | 304.2 (+3.34) | 84.4 (-1.07) | hybrid sensor kinase/response regulator |
| 2381 | 138.9 (1) | 574.4 (+4.13) | 127.1 (-1.09) | TCS, response regulator |
| 2382 | 186.2 (1) | 538.8 (+2.89) | 167.9 (-1.10) | TCS, sensor kinase |
| 2384 | 16.4 (1) | 1765.6 (+107.3) | 13.0 (-1.25) | tryptophan-rich sensory protein |
| 2560 | 20.8 (1) | 69.0 (+3.3) | 24.5 (+1.17) | aromatic alcohol reductase |
| 2562 | 29.4 (1) | 6016.0 (+204.3) | 28.1 (-1.04) | fascicin domain-containing protein |
| 3627 | 51.8 (1) | 149.4 (+2.8) | 46.2 (-1.12) | YceA |
| 3628 | 82.7 (1) | 258.1 (+3.1) | 70.3 (-1.17) | CybB, cytochrome b |
| 3629 | 430.5 (1) | 1684.3 (+3.9) | 422.8 (-1.01) | YceB |
| 3695 | 20.4 (1) | 68.3 (+3.3) | 22.5 (+1.10) | response regulator |
| Downregulated in KJΔChrR | | | | |
| 0227 | 256.49 (1) | 64.54 (-3.9) | 223.5 (-1.1) | MFS transporter |

^a^TPM, Transcripts Per Kilobase Million

^b^Negative fold changes represent genes that were significantly downregulated compared to wild-type KJ, whereas positive fold changes represent upregulation compared to wild-type KJ.

**Table S3. Expression of the PG homeostasis-associated genes in wild-type KJ and KJΔChrR revealed by transcriptome analysis.**

| Locus | Protein | TPM^a^ | | Fold change^b^ |
| --- | --- | --- | --- | --- |
|  |  | **KJ** | **KJΔChrR** |  |
| (A) Genes involved in PG homeostasis | | | | |
| PG biosynthesis pathway | | | | |
| Smlt0753 | MraY | 210.27 | 176.72 | -1.19 |
| Smlt0755 | MurG | 265.37 | 210.71 | -1.26 |
| Smlt3826 | PBP1a | 110.38 | 107.38 | -1.03 |
| Smlt3681 | PBP1b | 89.49 | 80.13 | -1.11 |
| Smlt3602 | PBP1c | 10.53 | 13.07 | +1.24 |
| Smlt4056 | PBP2 | 37.37 | 36.53 | -1.02 |
| Smlt0750 | PBP3 | 216.73 | 171.10 | -1.26 |
| Smlt0462 | PBP4 | 10.21 | 10.53 | +1.03 |
| Smlt4050 | PBP6 | 498.99 | 409.67 | -1.21 |
| PG metabolism pathway | | | | |
| Smlt0154 | Amidase | 107.04 | 111.80 | +1.04 |
| Smlt0155 | MltA | 56.70 | 61.57 | +1.09 |
| Smlt4052 | MltB1 | 336.04 | 284.12 | -1.06 |
| Smlt4650 | MltB2 | 63.15 | 76.71 | +1.21 |
| Smlt0994 | MltD1 | 218.85 | 249.93 | +1.14 |
| Smlt3434 | MltD2 | 167.35 | 174.52 | +1.04 |
| Smlt4007 | Slt | 179.05 | 170.41 | -1.05 |
| Smlt0412 | AmpN | 110.58 | 126.83 | +1.15 |
| Smlt0413 | AmpG | 52.40 | 57.15 | +1.09 |
| Smlt1245 | Opp | 59.56 | 69.53 | +1.17 |
| PG recycling pathway | | | | |
| Smlt3538 | NagZ | 108.91 | 116.08 | +1.07 |
| Smlt1562 | AmpDI | 34.71 | 36.13 | +1.04 |
| Smlt0415 | AnmK | 51.79 | 44.32 | -1.05 |
| Smlt0584 | MupP | 164.09 | 174.18 | +1.06 |
| Smlt1131 | AmgK | 58.41 | 58.35 | +1.00 |
| Smlt1130 | MurU | 39.73 | 43.63 | +1.10 |
| Smlt4020 | NagA1 | 74.97 | 69.76 | -1.07 |
| Smlt4438 | NagA2 | 37.56 | 38.19 | +1.02 |
| Smlt3414 | GlmM | 188.14 | 240.41 | +1.28 |
| Smlt4099 | GlmS | 227.01 | 217.65 | -1.04 |
| Smlt4108 | GlmU | 65.41 | 64.98 | -1.01 |
| Smlt3885 | Mpl | 105.06 | 98.95 | -1.06 |
| Smlt1119 | MurA | 226.48 | 216.16 | -1.05 |
| Smlt2127 | MurB | 173.79 | 147.91 | -1.17 |
| Smlt0756 | MurC | 363.09 | 274.34 | -1.31 |
| Smlt1170 | MurD | 116.21 | 105.44 | -1.09 |
| Smlt0751 | MurE | 256.82 | 220.21 | -1.16 |
| Smlt0752 | MurF | 258.57 | 210.95 | -1.21 |
| (B) β-lactamase genes | | | | |
| Smlt2667 | L1 | 27.08 | 25.21 | -1.07 |
| Smlt3722 | L2 | 56.44 | 27.18 | -2.08 |

^a^TPM, Transcripts Per Kilobase Million

^b^Negative fold changes represent genes that were significantly downregulated compared to wild-type KJ, whereas positive fold changes represent upregulation compared to wild-type KJ

**2.0**

**1.5**

**1.0**

**0.5**

**0**

**Relative β-lactamase activity**

**KJΔChrR**

**KJΔRpoEc**

**KJ**

**KJΔChrRΔRpoEc**

**KJΔChrRΔChrA**

**Fig. S1. CAZ-induced β-lactamase activities of *S. maltophilia* KJ and its derived mutants.** Overnight cultures of bacterial cells tested were subcultured into fresh LB broth with an initial OD_450nm_ of 0.15. After 3-h culture, CAZ at 1/4 MIC was added and further cultured for 0.5 h. Beta-lactamase activity was determined. The relative β-lactamase activity was normalized using the β-lactamase activity of KJ cells as 1. Bars represent the average values from three independent experiments. Error bars indicate the standard deviations for three triplicate samples.

**C23O activity (Uc/OD_450nm_)**

**None MD CAZ**

**Fig. S2. Impact of MD and CAZ on the expression of *rpoEc-chrR-chrA* operon.** Overnight culture of KJ(pRpoEc_xylE_) was subcultured into fresh LB broth (with or without the additives as indicated) at an initial OD_450nm_ of 0.15. The concentrations of MD and CAZ added were 16 μg/mL and 50 μg/mL, respectively. After 5-h culture, the C23O activity was measured. One unit of C23O activity (Uc) was defined as the amount of C23O that converted 1 nmol of catechol per min. The C23O specific activity was expressed as Uc/OD_450nm_. Bars represent the average values from three independent experiments. Error bars indicate the standard deviations for three triplicate samples.

**KJ**

**KJΔChrR**

**KJΔChrRΔRpoEc**

******

*****

**Relative transcript**

******

******

***rpoEc smlt2374 smlt2382 cytB L1 L2***

**Fig. S3. Validation of transcriptome results of wild-type KJ, KJΔChrR, and KJΔChrRΔRpoEc.** DNA-free RNA was isolated from KJ, KJΔChrR, and KJΔChrRΔChrEc, and reverted to cDNA using random primers. The transcripts of *rpoEc*, *smlt2374*, *smlt2382*, *cytB*, *L1*, and *L2* were determined by RT-qPCR. Relative transcript was calculated using the 2^-^*^ΔΔCT^* method using wild-type KJ transcript as 1. Data are the means from three independent experiments. Bars represent the average values from three independent experiments. Error bars represent the standard deviation for triplicates. *, *P* < 0.05, significance is calculated by Student’s *t*-test.
